# Testing and Analysis of Ultra-High Toughness Cementitious Composite-Confined Recycled Aggregate Concrete under Axial Compression Loading

**DOI:** 10.3390/ma16196573

**Published:** 2023-10-06

**Authors:** Li He, Sheng Peng, Yong-Sheng Jia, Ying-Kang Yao, Xiao-Wu Huang

**Affiliations:** 1 Hubei Province Key Laboratory of Systems Science in Metallurgical Process, Wuhan 430065, China; emp-heli@hotmail.com; 2 State Key Laboratory of Precision Blasting, Jianghan University, Wuhan 430056, China; 3 Hubei Key Laboratory of Blasting Engineering, Jianghan University, Wuhan 430056, China

**Keywords:** ultra-high toughness cementitious composite (UHTCC), recycled aggregate concrete (RAC), scanning electron microscope (SEM), confinement, engineering practice

## Abstract

In order to analyze the axial compressive properties of ultra-high-toughness cementitious composite (UHTCC)-confined recycled aggregate concrete (RAC), a batch of UHTCC-confined RAC components was designed and manufactured according to the requirements of GB/T50081-2002 specifications. After analyzing the surface failure phenomenon, load-displacement curves, scanning electron microscope (SEM), and parameter analysis of the specimen, the result shows that UHTCC-confined RAC is an effective confinement method, which can effectively improve the mechanical properties and control the degree of surface failure of RAC structures. Compared with the unconfined specimen, the maximum peak load of the UHTCC confinement layer with a thickness of 10 mm and 20 mm increased by 44.61% and 79.27%, respectively, meeting the requirements of engineering practice. Different fiber mixing amounts have different effects on improving the mechanical performance of RAC structural. The specific rule was steel fiber (SF) > polyvinyl alcohol fiber (PVAF) > polyvinyl alcohol fiber (PEF) > no fiber mixture, and the SF improves the axial compression properties of UHTCC most significantly. When there are strict requirements for improving the mechanical properties of the structure, SF should be added to UHTCC. On the contrary, PVAF should be added to UHTCC.

## 1. Introduction

Recycled aggregate concrete (RAC) is a green building material that has the characteristics of reducing carbon emissions, reducing environmental pollution, and saving nonrenewable natural resources in green buildings [1]. It helps to expand the utilization of building solid waste, reduce natural aggregate consumption, and help solve environmental problems. It is an important way to achieve the sustainability of building structures [2]. As the service duration increases, RAC structures will experience different levels of performance degradation and attenuation because of environmental erosion, natural disasters, and functional changes.

For RAC structures whose performance cannot meet the requirements of the specifications, conventional repair methods such as the enveloped steel jacket repair method [3], the carbon fiber reinforced polymer (CFRP) repair method [4], and the enlarging section method [5] have been adopted and used. However, ESJ has certain limitations in marine green buildings, and it is extremely difficult to meet the requirements for anti-corrosion treatment and chloride ion erosion prevention. CFRP has the advantages of being lightweight, high-strength, convenient construction, good corrosion resistance, and durability. However, the organic binder used in the material-to-material interface constraint will exhibit defects such as aging, poor high temperature resistance, and fire resistance when exposed to extreme conditions such as ultraviolet, humidity, high temperature, or fire for a long time, exposing the shortcomings of CFRP repair methods [6]. The enlarging section method requires sufficient curing time; otherwise, it cannot meet the strength requirements. The mechanization level of the enlarging section method is relatively low, and the labor cost is high. Ultra-high-toughness cementitious composite (UHTCC) is a special cement-based material added with a certain volume of specific chopped fibers, which can effectively reduce the erosion of chloride ions and other harmful examples on the steel inside the structure [7], change the failure mode of concrete single cracks, and have obvious advantages in the collaborative crack resistance of mortar base materials and fiber grids, making it an important repair method in the field of green building reinforcement. UHTCC show significant strain hardening and excellent crack resistance under both tensile and bending loads. There have been many studies on the mechanical properties of beams, columns, joints, and frame structures confined with UHTCC, revealing the reinforcement mechanism and mechanical laws of the confined components and structures [8,9,10,11,12,13]. Li et al. [14] studied the confinement effect of spraying a 20 mm thin layer of UHTCC at the bottom of a cracked concrete beam, which increased its ultimate bearing capacity by 117.5%. After confinement, the crack width was controlled below 0.1 mm, which was beneficial for improving durability. Kim et al. [15,16] studied the crack’s propagation mechanism at the bonding interface between sprayed UHTCC and existing concrete and conducted bending tests on beam components composed of equal-thickness sprayed UHTCC and concrete thin plates. They found that sprayed UHTCC significantly improved the bearing capacity of the composite components and had good crack control ability. Lim et al. [17,18] conducted experiments on UHTCC/concrete T-shaped incision composite beams and found that the UHTCC repair layers have better crack dispersion ability than the repair layers of steel fiber concrete and concrete. Xu Shiniang’s team [19,20,21,22,23] conducted bending performance tests on UHTCC-confined concrete composite beams. Zhang et al. [17,24] used UHTCC/concrete composite tensile testing to test the interface bonding tensile behavior between UHTCC and concrete. Zhang et al. [25] found that the number of microcracks in the UHTCC repair layer was related to the length of the UHTCC/concrete stripping interface. Kamada et al. [26] pointed out that in the confinement of defective concrete, decreasing interface roughness can make the performance of the UHTCC repair layer more outstanding. Kim et al. [15,27] studied the bonding behavior between sprayed UHTCC and concrete and found that the effect of sprayed UHTCC and cast-in-place UHTCC was the same. Wang et al. [28] conducted splitting tensile and shear tests on a total of 256 cubic bonding specimens to study the bonding behavior between UHTCC and existing concrete. Some factors, including the interface roughness, compressive strength, interface moisture states of existing concrete, and UHTCC pouring position, were investigated. Jiang et al. [29] focused on the mechanism of strengthening concrete columns with FRP grids/FCC using polyethylene-type FCC as the substrate. The experimental study was conducted on a standard concrete cylinder strengthened by an FRP grid/FCC, where new FCC material served as the matrix. The testing variables were plain concrete strength and different textile grids, i.e., basalt fiber reinforced polymer (BFRP) and CFRP grid. The uniaxial compressive performance was studied. The confinement of green building structures is essentially the confinement of one side of the interface between UHTCC and RAC, rather than wrapping the RAC to form a complete confinement mode. In order to provide a basis for the load design of the UHTCC-confined RAC interface under axial compression load, it is extremely important to study the bonding behavior between the UHTCC and RAC interface by referring to the research results of the UHTCC and concrete bonding behavior [30]. Therefore, it is still necessary to supply and complete the research on the axial compressive mechanical behavior of UHTCC-confined RAC.

The factors that affect the interface of UHTCC-confined RAC include two parts: UHTCC and RAC. The research on the influential mechanism of green building structures that meet the requirements of design specifications was relatively mature, so the research on the impact mechanism of UHTCC was mainly focused on. Taking full account of the influence of UHTCC fiber type and reinforcement layer thickness, axial compression tests and stress analysis were carried out on UHTCC-confined RAC to study its obvious failure phenomenon, load displacement curve, scanning electron microscope (SEM), and parameter effects. The dispersion and existence states of different fiber types in the matrix were obtained by SEM, revealing the influence of the UHTCC protective layer and fiber type on the compression performance of RAC.

## 2. Test Overview

### 2.1. Materials

During preparation, both the Hobart and cement paste mixers were used for UHTCC and RAC slurry mixing. In this study, the PI 42.5 reference Portland cement according to Chinese standard (GB8076-2016) [31], tap water, low calcium fly ash, medium sand (the particle grading conforms to Class II grading zone), gravel (produced by Hebei Yueshan Environmental Protection Technology Co., Ltd.), polycarboxylate superplasticizer (VIVID-651, produced by Guizhou Hengfan New Technology Development Co., Ltd.), and recycled coarse aggregate (produced by Hangzhou Ruichen Building Materials Co., Ltd.) were used to prepare RAC paste. The mix proportion of RAC is cement (kg/cm^3^):water (kg/cm^3^):medium sand (kg/cm^3^):gravel (kg/cm^3^):low calcium fly ash (kg/cm^3^):polycarboxylate superplasticizer (kg/cm^3^):recycled coarse aggregate (kg/cm^3^) = 1.0:0.89:2.29:5.26:0.74:0.04:2.29. The density is 2400 kg/cm^3^, the water-binder ratio is 0.89, the replacement rate of recycled coarse aggregate is 50%, the strength grade of RAC is C40, and the measured value of standard cubic axial compressive strength is 39.2 MPa.

**Figure 1:**
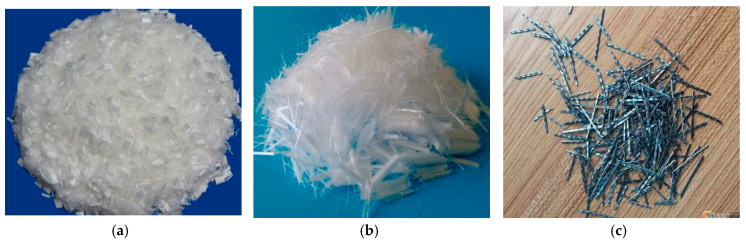
Appearance and shape of three fibers. (**a**) Polyvinyl alcohol fiber (PVAF); (**b**) Polyethylene fiber (PEF); (**c**) Steel fiber (SF).

While for the UHTCC paste, the binder materials include PI 52.5 Portland cement according to Chinese standard (GB175-2017) [32], tap water, medium sand (the particle grading conforms to Class II grading zone), polycarboxylate superplasticizer (VIVID-651, produced by Guizhou Hengfan New Technology Development Co., Ltd.), and low calcium fly ash (produced by Tianjin Zhucheng New Material Technology Co., Ltd.) were used instead. The mix proportion of UHTCC is cement (kg/cm^3^), low calcium fly ash (kg/cm^3^), medium sand (kg/cm^3^), water (kg/cm^3^), and polycarboxylate superplasticizer (kg/cm^3^) = 1.0:4.0:1.0:1.1:0.04. The density is 2500 kg/cm^3^, and the water-binder ratio is 1.1. Polyvinyl alcohol fiber (PVAF), polyethylene fiber (PEF), and steel fiber (SF) that meet Chinese standards were used and shown in Figure 1.

Moreover, add 2.5% volume content of fibers to the UHTCC slurry to ensure fluidity [33]. The length, diameter, length-width ratio, density, tensile strength, and elastic modulus of PVAF are 12 mm, 0.041 mm, 387.1, 1.3 g·cm^3^, 1560 MPa, and 41 GPa, respectively. The length, diameter, length-width ratio, density, tensile strength, and elastic modulus of PEF are 0.0068 mm, 0.5 mm, 324.8, 0.941~0.965 g·cm^3^, 21~38 MPa, and 0.84~0.95 GPa, respectively. The length, diameter, length-width ratio, density, tensile strength, and elastic modulus of SF are 30 mm, 0.32 mm, 75, 7.85 g·cm^3^, 86.5 MPa, and 80~90 GPa, respectively.

### 2.2. Specimens Design

The size of the specimen in the axial compression strength tests is 150 mm × 150 mm × 150 mm according to Chinese standard (GB/T50081-2002) [34], including 7 sets of 3 specimens in each group, totaling 21 specimens. The experimental variables include the reinforcement layer thickness and fiber type of UHTCC. The reinforcement layer thicknesses of UHTCC are 0, 10 mm, and 20 mm. The fiber types of UHTCC include PVAF, PEF, and SF. The numbering and configuration details of the test specimens are shown in Table 1.

### 2.3. Test Steps and Process

**Figure 2:**
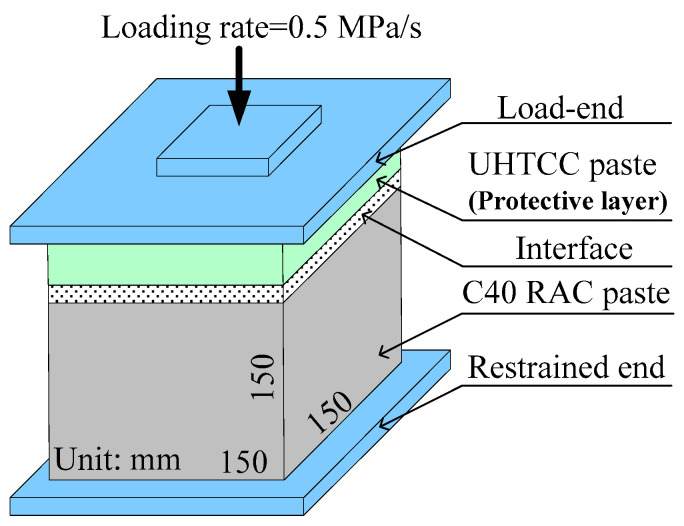
Loading process of the axial compression test.

As shown in the loading process in Figure 2, the axial load is provided by the WAW-E series microcomputer-controlled electro-hydraulic servo universal testing machine (Jinan Zhongluchang Testing Machine Manufacturing Co., Ltd.), which meets the standards of GB/T16826-2008, GB/T228-2010, and GB/T7314-2005 [35,36,37]. The loading rate should be controlled at 0.5 MPa/s until it is destroyed. The specimens from top to bottom are UHTCC, interface layers, and RAC. During the test, it is necessary to apply a uniformly thin layer of Vaseline to the upper and lower surfaces of the specimen. Collect damaged specimens, polish the surface, mark, and scan with SEM to obtain the interface microstructure of the fragments.

## 3. Analysis of Test Results

### 3.1. Failure Analysis

**Figure 3:**
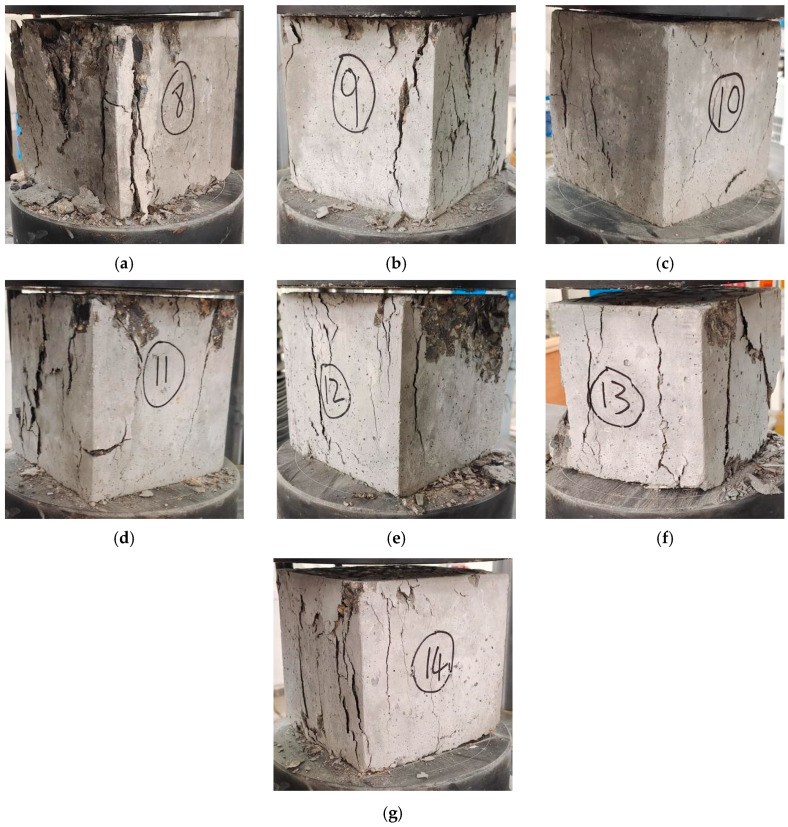
Diagram of failure mode. (**a**) Specimen C40; (**b**) Specimen C40-PVAF-10; (**c**) Specimen C40-PVAF-20; (**d**) Specimen C40-PEF-10; (**e**) Specimen C40-PEF-20; (**f**) Specimen C40-SF-10; (**g**) Specimen C40-SF-20.

Whether the fiber type and reinforcement layer thickness of UHTCC enhance the resistance levels of RAC to external forces needs to be determined based on the failure mode and failure rule of UHTCC-confined RAC under axial compression. As shown in the apparent failure phenomenon in Figure 3, the RAC failure mode of C40 strength degree has no special characteristics under axial compression, only forming vertical cracks and crushing at both ends. The failure degree of the specimen C40 is the highest, indicating that the UHTCC repair method is a good method to improve mechanical properties. For the degree of apparent failure, specimens C40-PVAF-10 < C40-PVAF-20, C40-PEF-10 < C40-PEF-20, and C40-SF-10 < C40-SF-20 indicate that, when the thickness reinforcement layer of UHTCC reaches 20 mm, the bearing properties of the specimen will be improved. The appearance of specimens C40-PVAF-10, C40-PVAF-20, C40-PEF-10, and C40-PEF-20 showed significant diagonal cracking, with specimen C40-PVAF-20 being the most prominent, indicating that both PVAF and PEF are beneficial for enhancing toughness and that PVAF is superior to PEF. The apparent specimens C40, C40-SF-10, and C40-SF-20 were mainly showed vertical penetrating cracking. From the perspective of cracking degree, specimens C40 > C40-SF-10 > and C40-SF-20 indicate that when the reinforcement layer thickness of UHTCC is 20 mm, the bearing properties of the structure can be effectively solved.

### 3.2. Micro-Structure Analysis

**Figure 4:**
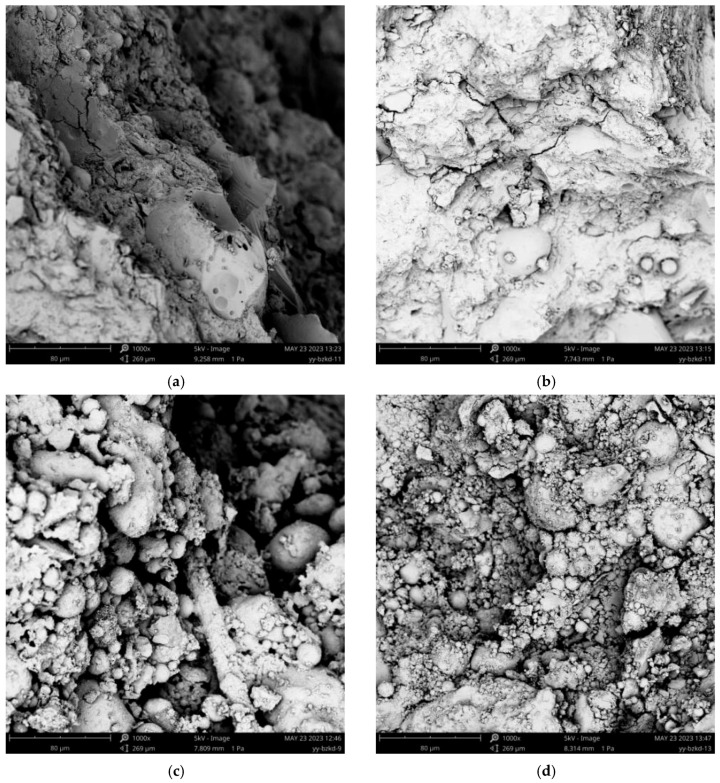

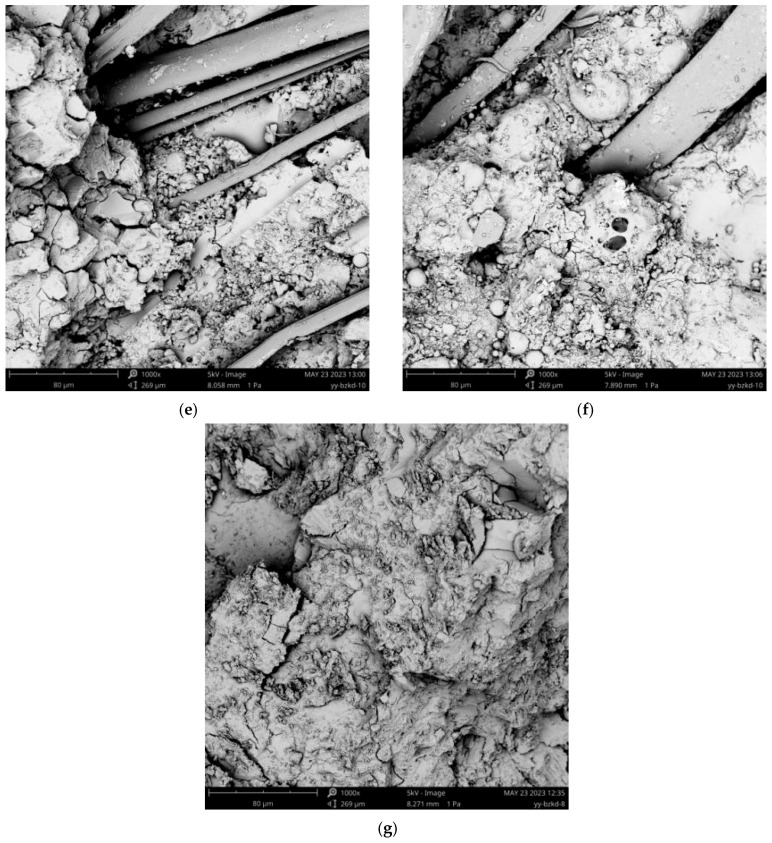
SEM of microstructure with three-type fibers.; (**a**) Specimen C40-PVAF-10; (**b**) Specimen C40-PVAF-20; (**c**) Specimen C40-PEF-10; (**d**) Specimen C40-PEF-20; (**e**) Specimen C40-SF-10; (**f**) Specimen C40-SF-20; (**g**) Specimen C40.

In order to analyze the micro-structure of UHTCC-confined RAC, a 1:80 μm measurement range was selected to conduct SEM of the specimen fragments after apparent failure identification. The internal defects of RAC can easily cause discontinuity or honeycomb shapes, forming discontinuous sponge-like distributions (as shown in Figure 4g), which is also the reason for the strong brittleness of RAC. The addition of PVAF can greatly enhance toughness; the cracks formed were suppressed in both width and length, and the number was also controlled. Moreover, the direction of the cracks was uncontrolled, and the formation of a mesh support structure was formed (as shown in Figure 4a,b), which significantly enhanced the bearing properties of the specimen. The addition of PEF basically maintained a very similar effect to the addition of PVAF, but the difference in slenderness ratio between the two resulted in the inability of PEF addition to form a stable network support structure, only forming a membrane structure (as shown in Figure 4c,d), which reduced the toughness in terms of reinforcement. The addition of SF can significantly increase frictional resistance and mechanical interaction (as shown in Figure 4e,f) and has a good inhibiting effect on crack development. Its action mechanism is similar to that between reinforcement bars and concrete, but it is extremely difficult to change the trend of crack development, resulting in a very similar apparent phenomenon in specimens C40, C40-SF-10, and C40-SF-20.

### 3.3. Mechanical Properties

**Figure 5:**
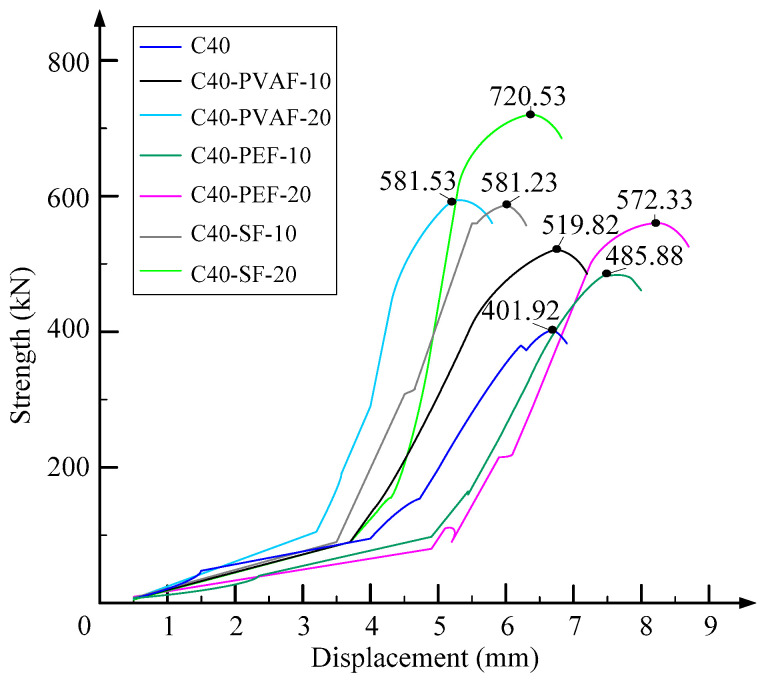
Load-displacement curves with three-type fibers.

Figure 5 shows the load-displacement curves of each specimen under an axial compression load. In terms of peak load, specimen C40 is the smallest, indicating that the UHTCC repair method is an effective reinforcement method. Comparing the load-displacement curves, it was found that the minimum displacement was required for the descending section of specimen C40, followed by specimens C40-SF-10 and C40-SF-20. There was no significant difference between specimens C40-PVAF-10, C40-PVAF-20, C40-PEF-10, and C40-PEF-20, indicating that the addition of PVAF and PEF had the most significant effect on toughness enhancement, followed by SF. Therefore, priority should be given to adding PVAF or PEF fiber materials to the UHTCC slurry.

### 3.4. Influence of UHTCC Protective Layers

**Figure 6:**
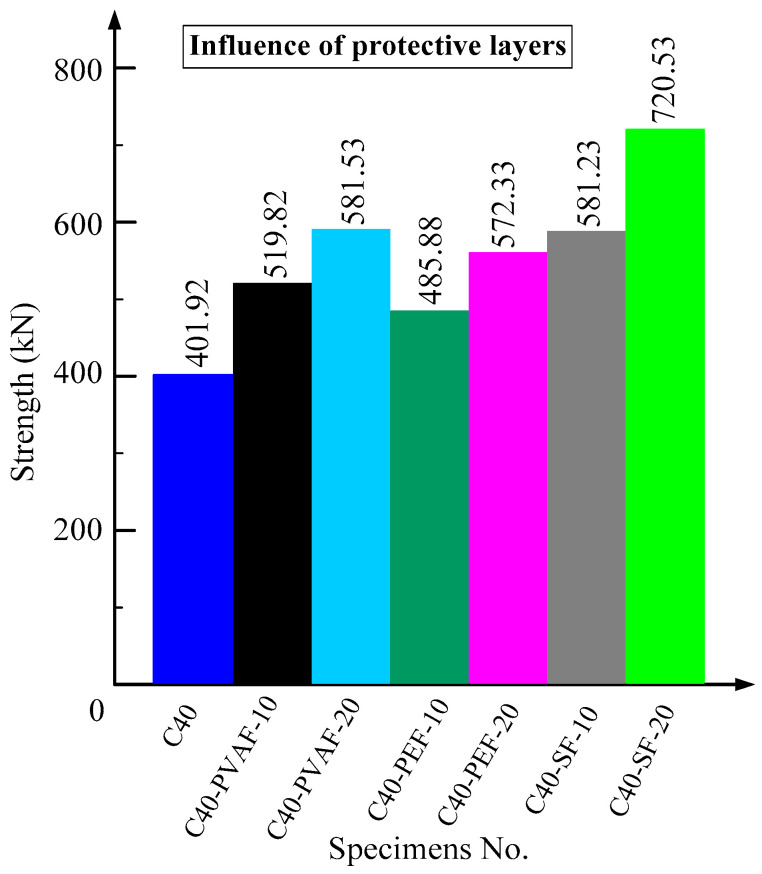
Influence of UHTCC protective layers.

Figure 6 shows the histograms of the peak load of each specimen under different UHTCC reinforcement layers. In this paper, the UHTCC reinforcement layers are set to 0, 10 mm, and 20 mm. When the UHTCC reinforcement layer is 0, the peak load is the smallest, followed by the specimen with a UHTCC reinforcement layer of 10 mm. The specimen with a UHTCC reinforcement layer thickness of 20 mm has the highest peak load, indicating that the greater the thickness of the UHTCC reinforcement layer, the better the improvement in mechanical properties. When the reinforcement layer thickness of UHTCC is 20 mm, the variation pattern of peak load is specimens C40-SF-20 > C40-PVAF-20 > C40-PEF-20. When the reinforcement layer thickness of UHTCC is 10 mm, the variation pattern of peak load was specimens C40-SF-10 > C40-PVAF-10 > C40-PEF-10. Compared with specimen C40, the peak loads of specimens C40-SF-20, C40-PVAF-20, and C40-PEF-20 increased by 79.27%, 44.69%, and 42.40%, respectively; the peak loads of specimens C40-SF-10, C40-PVAF-10, and C40-PEF-10 increased by 44.61%, 29.33%, and 20.89%, respectively. The addition of SF to UHTCC can significantly increase the axial compression mechanical properties of RAC. The thickness reinforcement layers of 10 mm and 20 mm can increase the axial force of the structure, which meets the needs of engineering practice. The order of the improvement effect on structural mechanics properties is PVAF > SF > PEF > no addition fiber.

### 3.5. Influence of UHTCC Fiber Types

**Figure 7:**
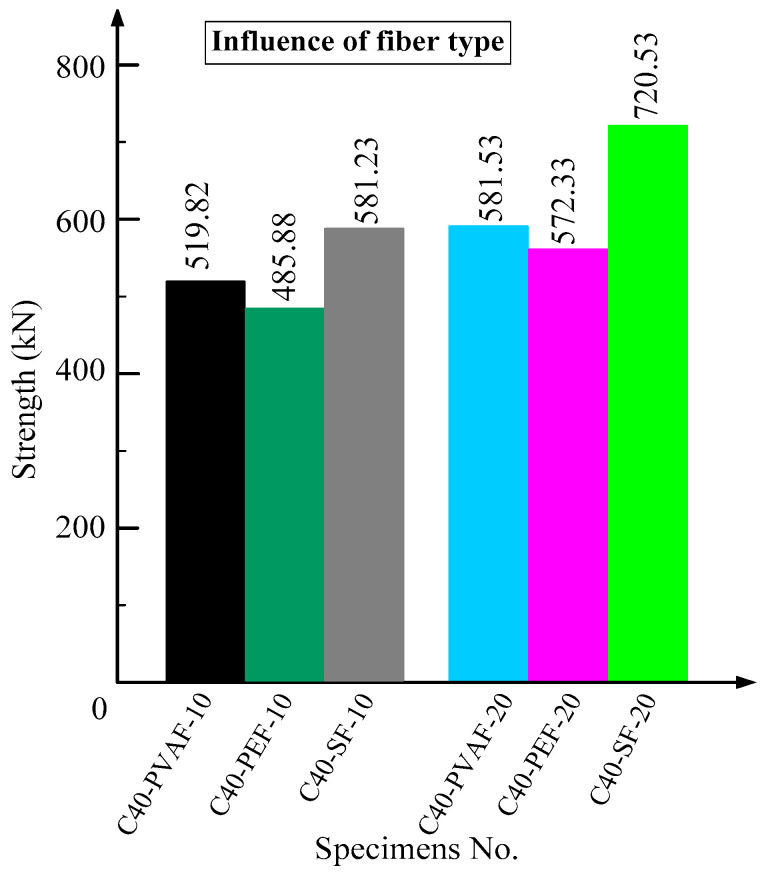
Influence of UHTCC fiber types.

Figure 7 shows the histograms of the peak load of each specimen under different types of UHTCC fibers. According to the different reinforcement layers of UHTCC, it can be divided into two groups: 10 mm and 20 mm. Compared with specimen C40-PEF-10, the peak load of specimens C40-SF-10 and C40-PVAF-10 increased by 19.62% and 6.99%, respectively. Compared with specimen C40-PEF-20, the peak loads of specimens C40-SF-20 and C40-PVAF-20 increased by 25.89% and 1.61%, respectively, indicating that the axial compression behavior of UHTCC with the addition of SF has the most significant improvement, but the improvement amplitude does not show a linear relationship with the increase in reinforcement layer thickness of the UHTCC. It is recommended that the UHTCC reinforcement layer have a thickness of 10 mm. If there is a high requirement for improving mechanical properties, it is recommended to use the addition of SF to UHTCC; otherwise, please use the addition of PVAF to UHTCC.

## 4. Summary and Conclusions

The influence of reinforcement layer thickness and fiber type on UHTCC-confined RAC cannot be ignored. Through a series of axial compression failure tests, the apparent failure phenomenon, load-displacement curves, SEM, and parameter effects of the specimens were obtained. The conclusions are as follows:

1. UHTCC reinforcement is an effective repair method. The mechanical properties of UHTCC-confined specimens have significantly improved, and the degree of apparent damage has been effectively controlled;
2. The variation pattern of the peak load is as follows: specimens C40-SF-20 > C40-SF20 > C40-PVAF-10 > C40-PEF-20 > C40-SF-10 > C40-PEF-10 > C40, indicating that the reinforcement layer thickness of UHTCC is 10 mm and 20 mm, respectively. This can effectively solve the bearing performance problem of RAC;
3. Compared with specimen C40, the peak loads of specimens C40-SF-20, C40-PVAF-20, and C40-PEF-20 increased by 79.27%, 44.69%, and 42.40%, respectively; the peak loads of specimens C40-SF-10, C40-PVAF-10, and C40-PEF-10 increased by 44.61%, 29.33%, and 20.89%, respectively. The addition of SF to UHTCC can significantly increase the axial compression mechanical properties of RAC. The order of the improvement effect on structural mechanics properties is PVAF > SF > PEF > no addition fiber;
4. Compared with specimen C40-PEF-20, the peak loads of specimens C40-SF-20 and C40-PVAF-20 increased by 25.89% and 1.61%, respectively, indicating that the axial compression behavior of UHTCC with the addition of SF has the most significant improvement, but the improvement amplitude does not show a linear relationship with the increase in reinforcement layer thickness of the UHTCC. It is recommended that a UHTCC reinforcement layer thickness of 10 mm;
5. If there is a high requirement for improving mechanical properties, it is recommended to use the addition of SF to UHTCC; otherwise, please use the addition of PVAF to UHTCC. Compared with PVAF and PEF. For improving the mechanical properties and 10 mm confinement layer, please add SF to UHTCC.

## Figures and Tables

**Table 1:** Specimen numbering and configuration details.

Specimen No.	Recycled Concrete Strength	Protective Layer Thickness/mm	Fiber Type	Peak Load/kN
C40	C40	0	-	401.92
	C40	0	-	
	C40	0	-	
C40-PVAF-10	C40	10	PVAF	581.23
	C40	10	PVAF	
	C40	10	PVAF	
C40-PVAF-20	C40	20	PVAF	720.53
	C40	20	PVAF	
	C40	20	PVAF	
C40-PEF-10	C40	10	PEF	485.88
	C40	10	PEF	
	C40	10	PEF	
C40-PEF-20	C40	20	PEF	572.33
	C40	20	PEF	
	C40	20	PEF	
C40-SF-10	C40	10	SF	519.82
	C40	10	SF	
	C40	10	SF	
C40-SF-20	C40	20	SF	581.53
	C40	20	SF	
	C40	20	SF	

## Data Availability

Some or all data, models, or code that support the findings of this study are available from the corresponding authors upon reasonable request.
